# A Study on the Relationship between Usability of GUIs and Power Consumption of a PC: The Case of PHRs

**DOI:** 10.3390/ijerph18041385

**Published:** 2021-02-03

**Authors:** José A. García-Berná, Sofia Ouhbi, José L. Fernández-Alemán, Juan M. Carrillo de Gea, Joaquín Nicolás, Begoña Moros, Ambrosio Toval

**Affiliations:** 1 Department of Computer Science and Systems, University of Murcia, 30100 Murcia, Spain; aleman@um.es (J.L.F.-A.); jmcdg1@um.es (J.M.C.d.G.); jnr@um.es (J.N.); bmoros@um.es (B.M.); atoval@um.es (A.T.); 2 Department of Computer Science and Software Engineering, UAE University, Al Ain 15551, United Arab Emirates; sofia.ouhbi@uaeu.ac.ae

**Keywords:** personal health records, energy efficiency, usability, environmental sustainability

## Abstract

Usability is key to achieve quality in software products. The client applications with a high score in usability might impact on the power consumption when they are run in a PC. For this reason, energy savings turn to be critical in green software systems. In this paper the relationship between the usability evaluations of the GUIs and the power consumption measurements of the main components of a PC were analysed. A set of 5 web-based personal health records (PHRs) were selected as a case study. The usability assessment was performed by an expert, employing the 14 principles of design by Alan Dix as heuristics. They were scored on a Likert scale after performing a collection of common tasks in the PHRs. At the same time, an equipment to measure the energy consumption of hard disk drive, graphics card, processor, monitor and power supply was used. Spearman’s index was studied for the correlations between the usability assessments and the power consumption measurements. As a results, some weak relationships were found. A total of 5 usability heuristics were observed to may influence energy consumption when they were considered in the implementation of the PHRs. These heuristics were the following ones: consistency, task migratability, observability, recoverability and responsiveness. Based on the results, the usability principles of design cannot always be related to lower energy consumption. Future research should focus on the tradeoffs between usability and power consumption of client applications when they are used in a computer.

## 1. Introduction

The efficient use of power in technology is driving the development of energy-saving architectures that require small amounts of power to work [1]. New approaches to evaluate energy efficiency are blooming in literature. Since software determines how hardware resources should be employed, the study of how both hardware and software perspectives affect power consumption is getting relevance lately [2].

The problem of inaccurate forecasting of the impact of ICT has been studied in literature [3]. Green technology becomes critical to avoid more damage to the environment. The link between ICTs and economic growth, energy consumption, and carbon dioxide emissions reduces environmental degradation in the medium and long run [4]. In this context, sustainable software can contribute in the reduction of resources waste. In fact, ICT have a significant impact on the levels of CO${}_{2}$ dumped into the atmosphere [5]. Greenhouse gas emissions (GHGE) produced by ICTs could increase to around a 14% of the total GHGE in 2016 [6]. Only from the ICT sector, 196 Mt CO${}_{2}e$ were thrown to the atmosphere in 2015 [7]. In this vein, sustainable software could reduce the power consumption from 30% to 90% [8].

Personal Health Records (PHR) are tools that allow people to store and check their state of health. These tools are managed by the users themselves [9], and have the potential to improve their wellness [10]. Patients and caregivers alike are enthusiastic about using these information systems (IS), and a rapid growth is observed in literature. However, PHRs are in their infancy [11]. There is a conceptual gap that can lead to confusion when defining the term PHR. Several similar terms name the aforementioned ISs intended for health data management [12]. Even though the benefits of PHRs are promising, research has proved that these systems are not widely adopted by the potential users [13]. Several possible reasons could explain this situation. System-related factors, such as perceived usefulness, health information understandability, personalization, and patient-clinician communication support, and user-related factors, such as social influence, self-efficacy, and willingness to share, were concerns in the intentions of using a PHR [14].

Personal information stored online is a major concern. In most cases users have no knowledge of where the information goes and how it is processed [15]. The eHealth tools can transform the industry, providing benefits like flexibility, cost and energy savings, resource sharing, and fast deployment [16]. On the other hand, the complexity of the healthcare systems, the abundance of information, incompatibilities between systems, and the lack of a unified framework create major challenges in information security [17].

In the healthcare sector often happens urgent situations where erratic accesses and exchanges of medical data between professionals and organizations may be carried out [18]. International regulatory bodies have pointed out the necessity for healthcare providers to protect themselves against cyber-attacks [19]. The security healthcare sector in the United States has been victim of a long path of cybercrime. More than 176 million patients have been affected by attacks on their medical data since 2009 [20]. According to the Protenus Breach Barometer, around 15 million records were accessed without authorisation in 2018. The aforementioned number of attacked records was the triple of the number of data breaches registered in 2017 [21]. According to the Department of Health & Human Services of USA, the number of compromised records had increased to around 42 million in 2019 [22]. Moreover, it is expected that around 20 million patients in the EEUU have been affected in the 10 biggest data breaches occurred during 2020 [23]. Thus, the protection of the medical data is a major issue to address since the number of security breaches is expected to be triple by 2021 [24].

Despite the popular belief that data breaches occur because of external hackers, in most cases the reasons stem from employee carelessness and/or information security policies and procedures that are poorly implemented [20]. Decreasing the security and privacy risks in the healthcare domain requires research on technological aspects alongside human aspects [25]. Several solutions have been proposed in literature so far. Concerning the technical side, the criptography to protect the communications and the storage of medical data is gaining popularity in the eHealth domain. As an example, blockchain technology is a promising solution to face privacy and security concerns [26,27,28]. The Internet of Things have the potential to change the physical interaction between the individuals and organizations, aiming at exchanging information in a secure and reliable way through IT infrastructure [29]. On the human side, preventive and corrective actions are needed to prevent health staff from causing security incidents [30]. In this vein, organisations should focus on security culture rather than organisational culture to improve information security awareness, saving time and resources [31]. Moreover, focusing on the workers can enhance information security, reducing the human error [32]. Educational programs to incorporate important concepts on cybercrime and cybersecurity are relevant. In addition, recommendations such as including material in the examinations, transition to practice modules, and hospital/community orientation programs are key to achieve the aforementioned goals [33].

The PHRs can help address several strands of the sustainability gap in public health systems [12]. The demand of healthcare could be decreased by providing preventive measures and self-treatment instructions to people [34], and tethered PHRs can also lower health costs by reducing unnecessary office visits and telephone calls [35]. They can be very useful in critical situations such as in the current COVID-19 pandemic, which has impacted the mobility of people and their access to healthcare resources. The ease of use has an impact on the intention of using a PHR [36]. In addition, connected health systems have the potential to contribute to the environmental sustainability [37]. ICT has two effects on environmental sustainability. On the one hand, the manufacturing processes can be very polluting. On the other hand, they can reduce energy needs when used for example in the healthcare systems [38]. It is worth noting that software itself can be developed to demand less energy [39]. The following research questions were posed for a better understanding of this study.

***RQ.1***  Is there a relationship between the evaluated usability of a GUI and the amount of energy spent in a PHR client application?
  ***RQ.1.1***  Can energy consumption be reduced by using a GUI with familiar elements that have been used before?
  ***RQ.1.2***  Can you contribute to the reduction of energy consumption by delegating parts of the tasks to the system?
  ***RQ.1.3***  Can you improve energy demands when there are no system crashes or processing interruptions?
  ***RQ.1.4***  Can energy consumption be reduced by avoiding repetitive data entry on the website?
  ***RQ.1.5***  Can energy consumption be lower when the system response time is short, allowing the task to be completed more quickly?

There is a lack of research on the interplay between usability and power consumption in ISs. As an example, users can employ a tool that has good usability characteristics more often and in a better way, as they find it more useful. On the one hand, an application can lead to a more energy demanding situation due to the graphic components that enhance usability. On the other hand, a good usability configuration of the web page may allow to complete a task quicker, requiring less amount of power. Therefore, a tradeoff between usability and power consumption must be studied for the development of ISs. To the best of our knowledge, no other study addresses this topic from an empirical perspective. Although some examples of sustainability and usability in eHealth can be found in literature, both aspects are approached in isolation in these works. For example, the communication of an Android application with blood glucose monitor devices over Bluetooth Low Energy was investigated, and found out that the digital certificates required by encrypted data led to usability challenges for patients and doctors [40]. In another example, expert-based usability evaluations of diabetes apps for iOS and Android were carried out, leaving out of the study the sustainability matters [41]. A reusable catalogue of sustainability requirements together with usability requirements for the development of mHealth applications was proposed. However, the relationships between both factors were not analyzed [42].

In this paper, the relationship between usability and power consumption in client applications was analysed. Statistical correlations were calculated between usability scores and power measurements. The goal was to uncover and explain potential couplings, such as if a client application with high usability assessments can spend less amount of energy at the same time. The significant correlations were illustrated with the graphical user interface (GUI) components that enhance usability, and the energy values of the hard disk drive, processor, graphics card, monitor and power supply (i.e., energy supplied to the computer). A set of representative tasks in PHRs were proposed. The tasks were performed over five PHRs while measuring the power consumption of the aforementioned PC components. At the end of each task a set of usability heuristics were scored on a Likert scale. The PHRs were chosen after following a rigorous literature review process performed twice, the first time in January 2018 and the second time in October 2020. No more PHRs were found for this study in the second iteration. Moreover, one of the PHRs had been shut down on November 2019. Despite the fact that the PHR was no longer available, the findings in the paper concerning the aforementioned PHR are important. They allow to raise awareness on the impacts that usability of the GUIs may have on the energy consumption of the main components of a PC when running client applications. It is worth noting that each PHR presented a particular functionality feature that stood out between the others [43]. Considerations to take into account in the client applications on usability and power consumption were particularly addressed in this work. The findings can be applied to other web portals such as social media, banking, newsletters, forums, etc.

This paper is based on a previous study on the same research topic [39]. Compared to the previous work, the present manuscript analyses the correlations between power consumption measurements and usability evaluations in PHR client applications. The Spearman’s coefficient was calculated for this purpose. As a result, correlations were found that, although weak, were significant. Of all the correlations calculated, a total of 5 usability heuristics stood out along with several PC components. It is worth noting that the heuristics were based on the usability principles proposed by the author Alan Dix [44]. The significant correlations were the following: (1) consistency along with the processor ($r_{s}=0.238,\;p=0.027$), monitor ($r_{s}=0.246,\;p=0.023$) and power supply ($r_{s}=0.254,\;p=0.019$); (2) task migratability along with the processor ($r_{s}=0.212,\;p=0.050$) and power supply ($r_{s}=0.237,\;p=0.028$); (3) observability with the monitor ($r_{s}=0.208,\;p=0.055$); (4) recoverability with the hard disk ($r_{s}=-0.279,\;p=0.009$) and graphics card ($r_{s}=-0.224,\;p=0.038$); (5) and finally, responsiveness together with the processor ($r_{s}=-0.268,\;p=0.013$) and power supply ($r_{s}=0.246,\;p=0.023$). The rest of the paper is organized as follows: in Section 2 the methodology followed in this study was described, separating between the selection process of the PHRs and the evaluation of these tools in terms of usability and energy consumption. In Section 3 the results of the statistical analysis between energy consumption and usability assessments were exposed together with the discussion of the results. Finally, the conclusions together with the future work were stated in Section 4.

### State of Art

User-centered design of PHRs plays an important role for achieving good acceptance among future customers. As an example, poor usability and functionality have resulted in a low utility, affecting enrollment and participation rates by both patients and clinicians alike [45]. Patients agreed that sharing medical information promotes a better healthcare service but are concerned about usability and privacy issues when accessing to these ISs [46]. Moreover, physicians anticipated resistance to use them by older patients and colleagues [47]. In addition, usability helps organizations in the customization of their ISs [48]. Several approaches to evaluate usability in e-health systems were used, including surveys, think-aloud protocol, cognitive walkthrough, usability experts evaluation, heuristics evaluation, persona-based inspection and task analysis.

A very common method to evaluate usability requirements is end-user surveys, contributing to user-centered design and increasing user satisfaction [49]. In another example, usability of an inpatient portal was analyzed via the think aloud protocol. Participants made operational errors with most in navigation and assuming non-existent functionalities. Usage learning styles varied, with age as a potential influential factor [50]. Cognitive walkthrough was also employed to detect usability problems that were improved with recommendations [51]. In addition, an experts evaluation of mobile applications for diabetics revealed that the best scored usability criterion was “comprehensibility”, while the lowest was “fault tolerance” [41]. Among 10 heuristics employed to evaluate the usability of e-health ISs exposed that “consistency and standards” was violated most frequently. Moreover, severity problems concerning “error prevention” and “help and documentation” were detected [52]. Based on the persona inspection, a remarkable finding exposed that the success of an electronic health record also relies on how usable the software is for clinicians [53]. In another example, an assessment based on task analysis showed that the difficulty to make errors was regarded as being 30% of overall usability. In addition, the factors of easiness to learn, efficiency to use and easiness to remember made up 20% each of overall usability. Finally, satisfaction only accounted for up to 10% of overall usability [54]. In general, methodological approaches to evaluate usability showed room to improve usability of PHRs [55].

The United Nations (UN), through its Sustainable Development Goal (SDG) 3, which is devoted to Health, maintain that “ensuring healthy lives and promoting the well-being at all ages is essential to sustainable development” [56]. Furthermore, Universal Health Coverage (UHC) is defined by the World Health Organization (WHO) as “ensuring that all people have access to needed health services (...) of sufficient quality to be effective while also ensuring that the use of these services does not expose the user to financial hardship”; UHC has become a priority for WHO in the last years and a powerful driver of health reforms in many countries [57]. According to WHO, eHealth in its different forms (e.g., mHealth, telehealth, electronic health records) is a key element in achieving UHC [58]. Besides, UN’s SDGs 12 and 13 are focused on Sustainable consumption and production and Climate change, respectively. Both emphasise the growing interest and concern of the society in general and the UN in particular for the environmental sustainability issues.

The intersection between environmental sustainability and software is twofold: Green in software and Green by software [59]. The former is focused on making software and the process of building software more environmentally sustainable, whereas the latter has to do with achieving more environmental sustainability by using software [60]. PHRs have the potential to cover both. They can be energy efficient and make healthcare systems more sustainable. It is worth noting, however, that the general framework of sustainable development, as defined by UN [61], includes the social and economic dimensions in addition to the aforementioned environmental dimension. Moreover, Goodland [62] also adds the human or individual dimension to the general analysis of sustainability. Finally, when it comes to the analysis of sustainability in the context of software and systems, another dimension (i.e., the technical dimension [63]) needs to be taken into account [64].

Reducing the energy costs of eHealth is another important factor to be addressed. Energy efficiency should be improved due to both, its expected intensive use, as well as the large amount of health data that could be accumulated in the future [65]. Energy consumption was considered a fundamental sustainability characteristic in PHR architectural design. Bearing this in mind, an adaptation of the Attribute-Driven Design method including sustainability as a driver was introduced. The aim was to design a sustainable architecture of a cloud-based PHR [66]. The blockchain technology is also interesting for electronic healthcare data exchange in a distributed environment [67]. Blockchains offer promising features when it comes to security and privacy in PHRs, although power consumption can be dramatically high owing to their energy demanding nature [68]. Several blockchain systems were studied to determine which implementation is more suitable for PHR applications, finding the one that incurred a lower energy consumption [69]. In other work, the size of the block was modified to minimize energy consumption in a continuous remote patient monitoring system [70]. Another example was found in a secure network protocol for a body sensor network connected to a PHR that was designed and evaluated, establishing communications that are energy efficient [71].

## 2. Materials and Methods

**Figure 1:**
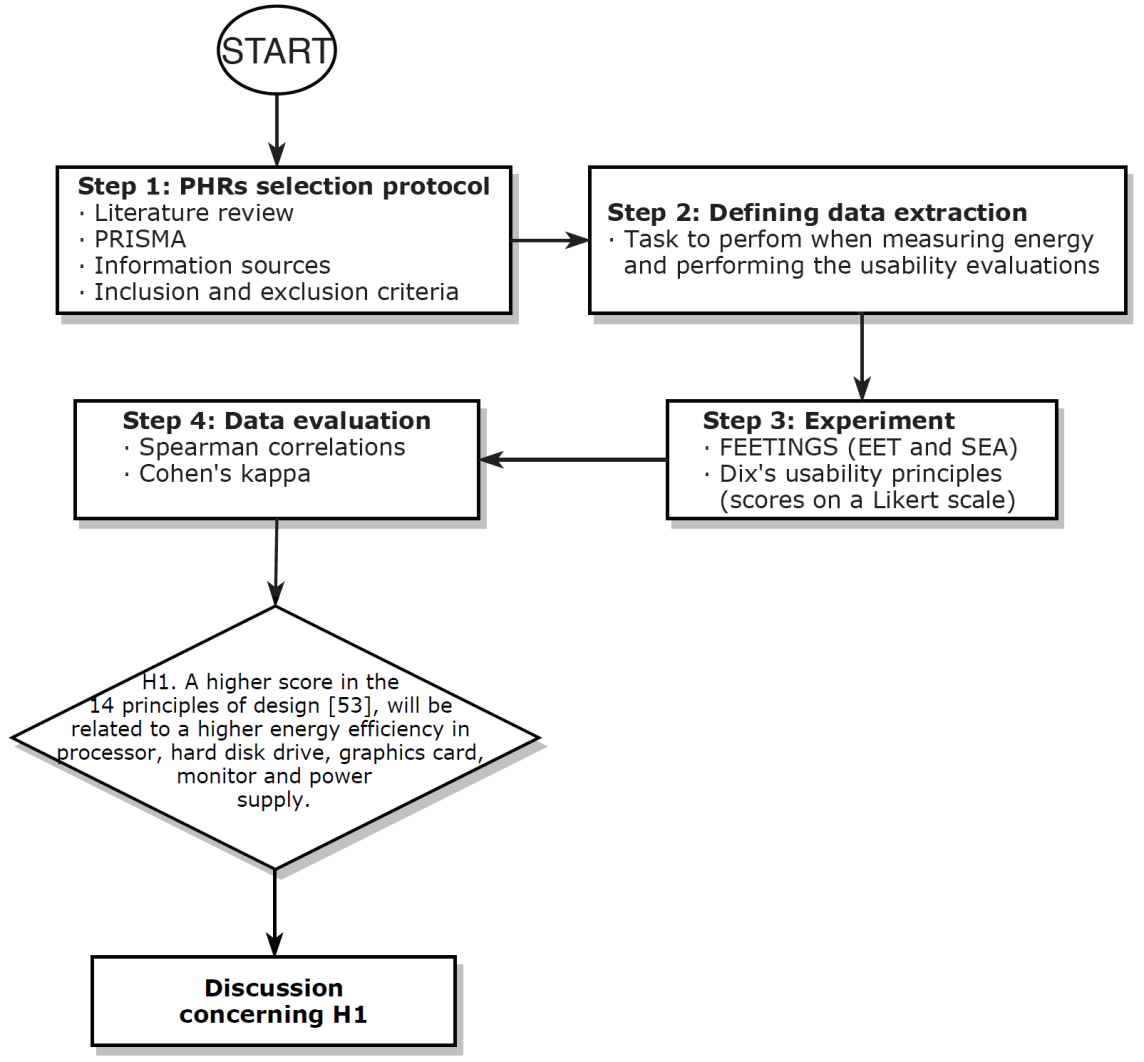
Research flow chart.

Figure 1 depicts the research protocol of the study. The steps performed in this research were: (i) defining the criteria to select the PHRs that should be considered, (ii) creating a framework to assess both energy consumption and usability of the PHRs, (iii) picking the device with which to collect the power data and choosing the usability assessment process, and finally, (iv) running the experiment and study the data with statistical analysis.

### 2.1. PHRs Selection

In this paper, free client applications were studied since they attract more attention than paid tools [72]. A protocol was followed for the selection of the aforementioned e-health applications. Based on a previous work [9], the guidelines proposed by the Preferred Reporting Items for Systematic reviews and Meta-Analysis (PRISMA) group [37] were applied. These recommendations allowed to guarantee that the search and the retrieval process was accurate and impartial. The search string “PHR providers“ OR “PHR website” was used in the ACM Digital Library, IEEE Digital Library, Medline and ScienceDirect databases. No restriction on the period of time was employed in the search. The obtained papers were read carefully to find available PHRs. An inclusion criterion (IC) pointed out that the PHR had to be in web-based format. A total of 19 PHRs were found with the aforementioned restrictions.

**Figure 2:**
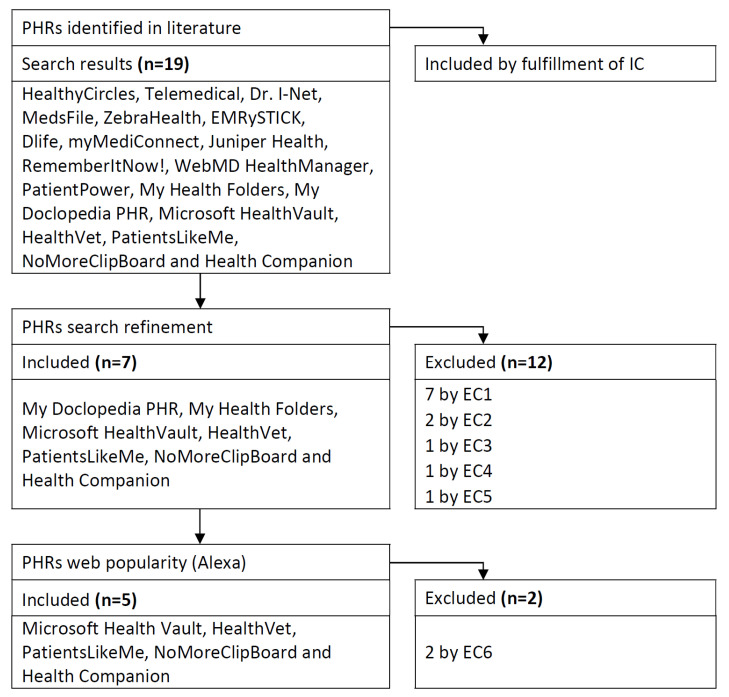
Prisma flow chart.

In addition, exclusion criteria (EC) were proposed to make the search for the PHRs more specific. They consist of the following ones: no longer available PHRs (EC1), requires payment (EC2), the inscription is not possible (EC3), malfunctioning (EC4), only available in a particular country (EC5), and low-popularity (EC6). The EC6 criterion was applied with the Alexa website [73]. Alexa online tool allowed to know the popularity of a webpage. The aforementioned web portal provides with a score concerning the estimated daily average of unique visitors to the website, and the estimated number of page views on the website. The estimates are based on data retrieved in the web servers of the United States or from all over the world. In the Alexa tool, the higher the value, the less popular a website is. Alexa score was set at more than 10 millions as a threshold to consider a low popularity of the portal. Both scores from the United States or from global servers were contemplated with the same importance. In fact, the highest score of the 2 that exceeded the threshold, determined the EC6 to be fulfilled. In some cases, the Alexa score was unavailable, which also led to a low popularity consideration for the web portal. The rest of the ECs were applied by an experts evaluation of the PHRs that fulfilled the IC (see Figure 2).

From the 19 PHRs selected initially, those that met ECs were discarded. Thus, a first rejection of PHRs was carried out. HealthyCircles, Telemedical, Dr. I-Net, MedsFile.com, ZebraHealth, EMRySTICK and Dlife were dropped due to EC1, myMediConnect and Juniper Health because of EC2, RememberItNow! by EC3, WebMD HealthManager by EC4, and PatientPower by EC5. Finally another round to discard more PHRs was carried out. In this case My Health Folders and My Doclopedia PHR fulfilled EC6. Both PHRs presented a popularity score higher than the threshold. General speaking, the Alexa website showed a very low popularity of the PHRs, except for HealthVet that stood out between the rest. Finally the PHRs selected were: HealthVet (Alexa punctuation of 858 in the U.S.; 4340 globally), PatientsLikeMe (U.S. Alexa punctuation of 31,540; global 96,217), Microsoft HealthVault (U.S. Alexa rank of 138,421; global 480,911), HealthCompanion (U.S. 1,703,097; global not available) and NoMoreClipBoard (U.S. 3,478,250; global not available).

The selection protocol was implemented in January 2018, and checked again in October 2020. The search for PHRs was repeated a second time. In the latter, the new literature was carefully read to find new PHRs available online. As a result, no more PHRs that met the inclusion and exclusion criteria were detected apart from those already selected. HealthVault was no longer available as of 20 November 2019. However, the energy consumption measurements, usability assessments and the inspection of the PHR according to the results were carried out before the aforementioned event. The findings obtained from the removed PHR were considered interesting to keep in the manuscript since they shed light to the research topic. Figure 2 shows the whole selection process of the PHRs [43].

The PHRs used in this research had at least one particular feature of functionality that stood out between the rest. A wide range of functionality expected in the PHRs was covered in this study because of the proposed selection protocol [74]. In Table 1, the particular features of functionality are shown. The information was extracted from the webpages of the PHRs. With this in mind, the study sample was representative in terms of the main functionalities that can be found in the PHRs.

### 2.2. PHRs Evaluation

The use of the PHRs was studied with a collection of representative tasks. Different scenarios were considered to capture the mental model of typical PHR users [75], and to better understand the correlations between GUI usability assessments and energy consumption measurements [76]. The tasks emerged from the point of view of two key roles, the patients and the healthcare staff. Both were selected to represent two profiles that complement each other. While patients may not know what a PHR is, health staff may be used to it and propose its use to patients [77]. In addition, health staff and patients can perform different tasks in the PHRs. The tasks tested in the PHRs were identified through common needs detected for better interaction between the selected profiles. In addition, the recommendations of the American Health Information Management Association (AHIMA) on the definition of a PHR were also taken into account in proposing the tasks [78]. The selection of the tasks was also based on previous work [9]. Table 2 shows the collection of the 20 common PHR tasks identified.

### 2.3. Usability Assessment

Usability features of the web portals were studied. An evaluation method based on experts’ opinions was applied together with heuristics evaluation. As stated in literature, a combination of different usability assessment techniques that compliment each other should preferably be used for a more powerfull results [79]. A set of 14 principles of design, known as the Dix principles [44], were employed as heuristics. A Likert scale was used in each one of the principles to analyze the usability, from 5 (very well supported) to 1 (very little supported). The principles of Dix were scored by an expert. The auditor has more than 4 years of experience in usability evaluations, and has been involved in several usability evaluations. The background is related to design of GUIs and usability of ISs. In addition, the auditor has a long history of giving lectures on GUIs and usability at the university. The usability evaluations were performed by the auditor right after finishing each one the tasks. All the Dix principles were scored using the defined Likert scale in each of the tasks proposed for the PHRs (see available Excel files online [80]). All the principles employed in the usability audit are described bellow.

- Predictability. The impact past interaction has on the ease of taking future actions.
- Synthesizability. Easiness to evaluate past operations on the current state.
- Familiarity. Previous knowledge and experience of users in other domains can be employed in a new system.
- Generalizability. Enhance specific interaction knowledge within and across applications to other similar situations.
- Consistency. Similar input–output behaviour in situations or task objectives that are the same.
- Dialog initiative. Independency in the input dialog of the system due to artificial constraints.
- Multi-threading. Possibility that the system allows user interaction in more than one task at a time.
- Task migratability. A task that when is performed can be internalized by the system or the user or done between both.
- Substitutivity. Input and output values that are equivalent can be substituted among themselves.
- Customizability. Easiness to modify GUIs by the user or the system.
- Observability. Easiness to check the internal state of the system concerning its perceptible representation.
- Recoverability. Provide with corrective actions in case of an error is made.
- Responsiveness. Support for users to check fluidity of the system when it is in operation.
- Task conformance. The system services cover all the tasks that users can perform and in a comprehensible manner.

### 2.4. Energy Expenditure Evaluation

**Figure 3:**
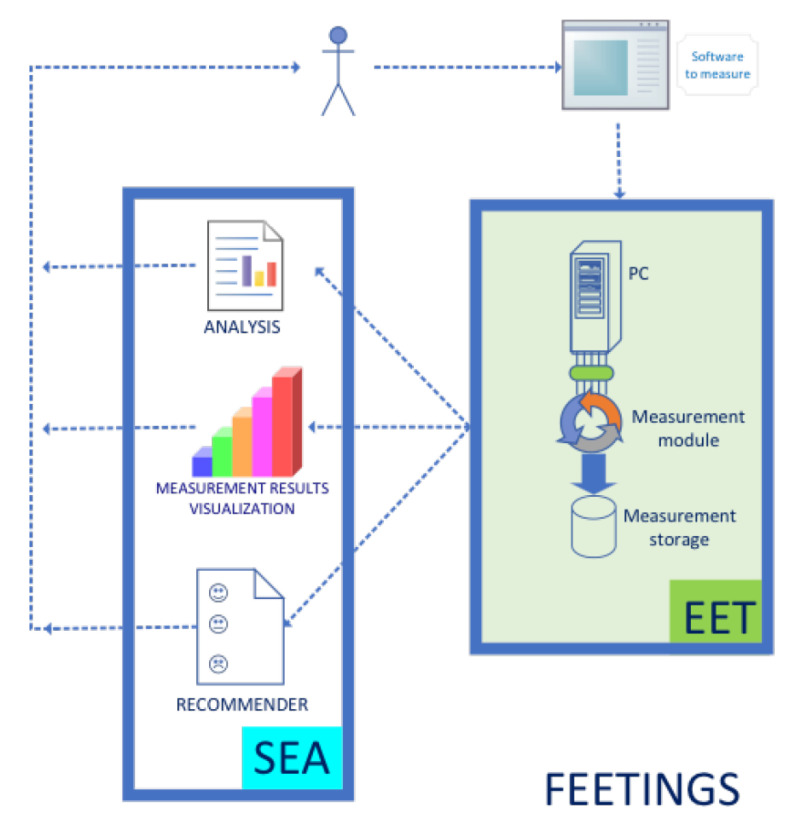
Whole FEETINGS system.

**Figure 4:**
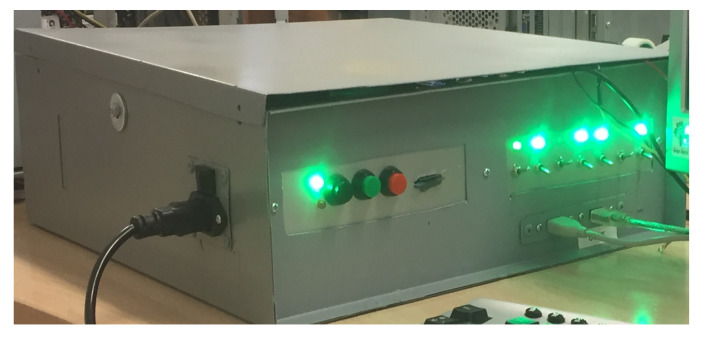
EET hardware.

The power spent by a piece of software may be extracted by direct measurements, simulation or estimates [81]. In this experiment, the power required by a host machine when performing the tasks in the PHRs was measured with a particular system. The Framework for Energy Efficiency Testing to Improve eNvironmental Goals of the Software (FEETINGS) [82] was employed for direct measurements. FEETINGS allows to measure and analyze the energy efficiency of a software application. This framework consists of the Energy Efficient Tester (EET) and the Software Energy Assessment (SEA) (see Figure 3 and Figure 4). EET is the core of the system and is provided with 5 probes attached to the main components of a PC. A total of 3 internal probes collect power data of processor, hard disk drive and graphics card. Moreover, a set of 2 external probes measure the energy required by the monitor and power supply (total energy consumption of the PC). This equipment is capable of providing instant power consumption in real time and in watts of the aforementioned components. The SEA subsystem manages the data generated by EET. The inputted values are processed and analysed for a proper presentation of the resulting information from the computations (i.e., mean values, standard deviation values, timestamps, number of samples, etc.). FEETINGS was especially useful for measuring power consumption when running a client application on a computer. Its portability and ease of use allowed to obtain power measurements from the host machine.

EET was connected to a computer provided with a thin film transistor liquid crystal display (TFT-LCD) monitor model Philips 170S6FS. The PC components were a GigaByte GA-8I945P-G motherboard, an Intel Pentium D @ 3.0 GHz processor, a set of 2 modules of 1 GB DDR2 @ 533 MHz RAM memory, a Samsung SP2004C 200 GB 7500 rpm hard disk drive, a Nvidia GeForce GTS 8600 graphics card, and a Aopen Z350-08FC 350 W power supply. The operating system installed in the PC was Microsoft Windows 7 Professional version. The navigator employed to perform the tasks in the PHRs was Chrome version 62. Moreover, background running processes were disabled as much as possible to avoid the appearance of unexpected overloading situations.

**Figure 5:**
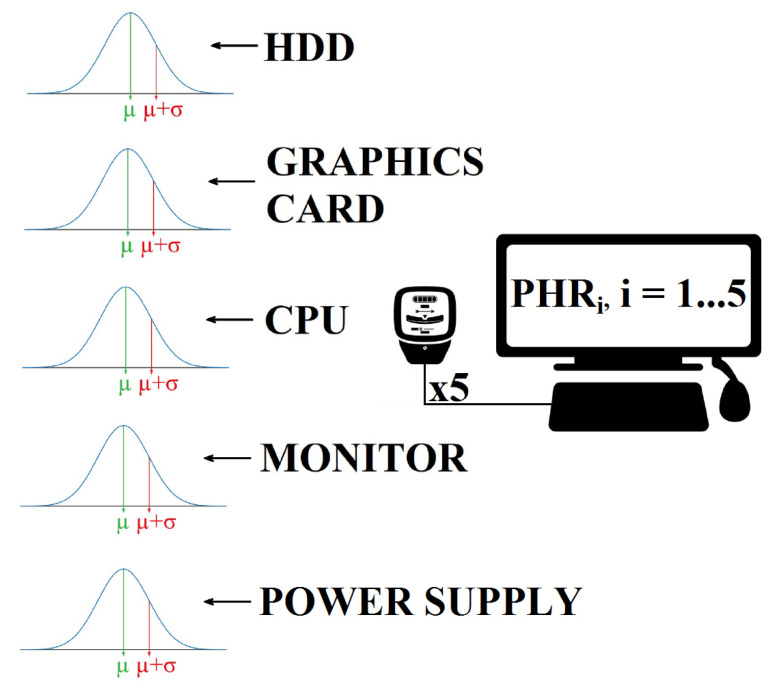
Process followed when measuring the power consumption.

Before starting with energy consumption measurements, the auditor synchronized with a colleague to perform the experiment. The colleague activated the EET system to collect power measurements at the same time that the task was started by the auditor. The proposed tasks were performed 5 times for each PHR. It is worth noting that the scan frequency of the equipment allowed to collect an important number of power measurements simply by doing the tasks 5 times. The aim of carrying out this number of iterations was to smooth the data and remove any possible outliers that may appear. For each sensor the mean value of power consumption was calculated from the measurements collected in each iteration. The task’s mean value of energy consumption was obtained with the 5 mean values from each sensor. Figure 5 gives an idea of the process followed.

### 2.5. Hypothesis

The topic of the paper is relevant given the alarming situation concerning energy waste, resource shortage, and pollution of the planet. The use of technology is widespread worldwide and its growth is unstoppable. As already mentioned, if good usability is implemented in client applications, energy efficiency could be boosted at the same time. As a result, contributing to improve the environmental situation would be possible. It is worth noting that, although the reduction of power wasted by an individual could be small, globally it is not [83]. The inherent replicability of software motivates the study presented in this manuscript. Concerning the research questions posed in Section 1, the following hypothesis was investigated. The aim was to evaluate if there is a relationship between power measurements and usability evaluations.

**Hypothesis 1** (**H1**)**.** *A higher usability score in the 14 principles of design [44], may be related to a lower power consumption in the processor, hard disk drive, graphics card, monitor and power supply.*

A total of 19 variables were defined: one variable per each principle of Dix with the usability evaluations, and one variable per each PC component with the power expenses. The sample size had a dimension of 100 formed by the 20 tasks executed in the 5 PHRs. For each task in each PHR the usability was assessed and the power consumption measured, generating a total of 19 values one per each variable. All the collected data extracted for the statistical analysis was distributed in a matrix, in which the 19 variables were placed in the columns and the 100 values of each variable in the rows. The data are presented in the input data tab of the file Results.xlsx [80].

Since the usability assessments were ordinal values while the power consumption figures were continuous, the Spearman’s correlation allowed to obtain the relationships between both variables. The calculation was performed with the resulting 70 pairs of combinations between the 19 columns, that is to say the 14 principles of Dix and the 5 PC components.

## 3. Results and Discussion

In this section the results are presented. The power measurements of the PC components together with the evaluations in usability when performing the tasks were saved in Data.xlsx. This file is also available online [80]. Moreover, the results of the non-parametric test are displayed in the Excel file Results.xlsx. The results of this section served to confirm or reject the hypothesis proposed in the previous subsection.

The Spearman’s Rank Correlation Coefficient $r_{s}$ and the *p*-value were calculated. The $r_{s}$ value is a statistical measure of the strength of a link or relationship between two sets of data, while the *p*-value is the probability of obtaining results as extreme as the observed results of a statistical hypothesis test, assuming that the null hypothesis is correct.

Significant correlations were found several times even though the relationship strengths were weak (see Table 3). That was the case of principle 5, Consistency, with the processor ($r_{s}=0.238,\;p=0.027$), monitor ($r_{s}=0.246,\;p=0.023$) and power supply ($r_{s}=0.254,\;p=0.019$). There was also a significant correlation between Principle 8, Task migratability, and processor ($r_{s}=0.212,\;p=0.050$) and power supply ($r_{s}=0.237,\;p=0.028$). Another important correlation was found in the case of principle 12, Recoverability, with the hard disk drive ($r_{s}=-0.279,\;p=0.009$) and the graphics card ($r_{s}=-0.224,\;p=0.038$). Finally, principle 13, Responsiveness, also arose significant correlations among the processor ($r_{s}=0.268,\;p=0.013$) and power supply ($r_{s}=0.246,\;p=0.023$). In order to give a whole picture of the relationship between usability evaluations and energy consumption more results were obtained. Apart from those aforementioned, the average usability score per task was computed with the values of all the heuristics (column DM in Excel file Results.xlsx [80]). The Spearman correlation was calculated together with the energy consumption values. As a result, no significant correlations were found. In Table 3 all the correlations between usability assessments and power consumptions are exposed. The whole data set with all the results can be retreived, downloading the file Results.xlsx.

Given the fact that only one auditor performed the usability assessment, a consistency analysis was carried out. The Split-Half Reliability coefficient was calculated with the usability evaluation of each PHR. As a result, the coefficients were in all the cases more than 0.80 (see Table 4).

As exposed in Table 3, several significant correlations were found between the score of the heuristics and the energy consumption of the PC components. An analysis in detail was carried out in each case where the correlation was significant. Moreover, the reasons of the extracted results were illustrated for a better understanding on how usability can impact on energy consumption. In the following subsections the principals and rationale of the most remarkable correlations found in the results were discussed.

### 3.1. Consistency

- ***RQ.1.1** Can energy consumption be reduced by using a GUI with familiar elements that have been used before?*

This design principle is one of the most widely applied in developing GUIs. Consistency within an application can reduce error rate and task completion time [84]. This principle of design relates to the likeness in behaviour arising from similar situations or similar task objectives [44]. Regarding the results, a more consistent system leads to more power consumption in processor ($r_{s}=0.238,\;p=0.027$), monitor ($r_{s}=0.246,\;p=0.023$) and power supply ($r_{s}=0.254,\;p=0.019$).

From the evaluation of the PHRs in Task 1, sign up, and Task 2, login, similar behaviours were expected, which is, accessing to the web portal for its use. HealthVault presented a high mark in consistency due to the fact that this action required a Microsoft^TM^ account [85] for registration. This security feature could be satisfactory for many users that previously had an email account at this domain. In addition, familiarity with past experiences is related to this principle [44]. Remarkable power expenses in these tasks were found for the processor in task 2, 3.4 W, for the monitor in task 1, 60.39 W and for the power supply in task 2, 185.57 W. This could be explained by the fact that to perform the sign up or login process, a two-step authentication is demanded. Moreover, screen changes or a more computational performance may demand more energy in these components [86].

### 3.2. Task Migratability

- ***RQ.1.2** Can you contribute to the reduction of energy consumption by delegating parts of the tasks to the system?*

In this principle, the easiness to delegate the task from the user to the system or the other way around is considered [44]. Task migratability is one of the most proposed usability principles in HCI studies [87]. Professionals must predict appropriate grades of automation in future situations and how to support them [88]. To this end, software solutions should allow users decide which activities to perform or automate [87]. A high level of task migratability can lead to reduce the energy consumption of the graphics card as confirmed in our experiment ($r_{s}=-0.191,\;p=0.079$). However, this feature may also have a negative impact on the power consumed by the processor ($r_{s}=0.212,\;p=0.050$) and power supply ($r_{s}=0.237,\;p=0.028$).

In PatientsLikeMe the autocomplete functionality was implemented when performing task 14, information of conditions search. This feature is particularly helpful when it comes to remember the names of illnesses, speeding up the search process [89]. Task migratability punctuation in the activity 14 was high in spite of the search field was small, impacting negatively on the usability of this feature [90]. The energy consumption of the power supply was also high (236.61 W), since extra energy consumption was required to autocomplete the names [91].

Task 19, exiting the PHR, generated the highest amount of power consumption in PatientsLikeMe. A total of 8.28 W were consumed by the processor and 281.45 W by the power supply. This particular amount of power need could be explained by the logout process in the PHR. In PatientsLikeMe there exist the possibility to logout from a single sign-on mode. This way to perform the task can lead to higher demands of power since more technical steps are necessary to logout [92]. Single sign-on systems have implications on usability [93], motivating its use [94] since productivity increases when the time needed for authentications is reduced [95]. Moreover, users leverage one single account to use multiple websites [96].

Migratability-aware features such as macros could generate a GUIs that is more efficient [97]. Nevertheless, no macros were found in any of the selected PHRs. Another way to improve task migratability is to optimize the server side. There are technologies such as Ajax which allows to shift part of the processing to the client [98]. This trend can be found in many web developments, thus increasing energy consumption of the applications.

### 3.3. Observability

- ***RQ.1.3** Can you improve energy demands when there are no system crashes or processing interruptions?*

Technology users always appreciate that the system works correctly at an internal level, without happening blockages or processing interruptions. Concerning the results of the experiment, a better observability entailed a high power demand of the monitor ($r_{s}=0.208,\;p=0.055$). This could be explained by the presence of graphic elements in the interfaces such as progressing bars and loading icons. Despite they can improve usability [99], an increasing of energy consumption may be produced [100]. In particular, progress bars can exhibit nonlinear behaviours such as acceleration and deceleration [101], impacting on the instant power consumption.

Scrollbars allow to know about the internal state of a computer whenever a user moves around in a running website. However, websites continue to feature poorly designed scrollbars [102]. Scrollbars were also observed in most of the PHRs. However, their use may lead to peak power consumption. In addition, scrollbars were proposed in literature to be removed for the design of more energy efficient GUIs [91]. In this sense, the number of screen updates involved in the interaction with the system is a key factor when it comes to power consumption [103]. Techniques such as a next page button can be employed to traverse a large section of a document, thus reducing the number of screen updates [104] and the consumed power [103].

### 3.4. Recoverability

- ***RQ.1.4** Can energy consumption be reduced by avoiding repetitive data entry on the website?*

This principle of design relates to the ability to reach a desired goal after making some errors in a previous interaction [44]. Recoverability has been recognized to favor positively the endurability of software systems [105]. Moreover, this non-functional property indirectly reflects the greenness of an application [106]. A high recoverability occurs when users feel free to explore on the system without a losing of the data [107]. Based on the results, the implementation of this principle generated low power demands on the hard disk ($r_{s}=-0.279,\;p=0.009$) and the graphics card ($r_{s}=-0.224,\;p=0.038$).

The lowest power consumption on the hard disk (14.249 W) appeared in HealthVault when performing task 4, check the profile. Moreover, this task received the highest punctuation in recoverability as well. Easy steps were carried out to correct the profile. It only took one click to access the profile modification page. The low power consumption on the hard disk could be explained by the combined use of cache memory and hard disk in the web portal. These mechanisms can significantly improve both energy efficiency [108] and performance [109], avoiding the long waiting time and high power consumption of hard disks’ spin-ups [110].

A high power consumption in graphics card was found in HealthVet when performing task 14, information of conditions search. This task generated several display changes together with a new tab opened in the navigator to perform the search. This led to a higher power necessities of the graphics card [104]. In addition, recoverability evaluation was very low due to the cumbersome process followed to access to this functionality in HealthVet. Conversely, the Veterans Health Library is accessible without login in HealthVet [111]. Typing directly the web link in the navigator turned to be simpler as well as more energy efficient since less steps were necessary to carry out. On the other hand, lots of quality images were placed in the Veterans Health Library, which forced a higher performance of the graphics card, and impacting direclty on the power consumption [91].

### 3.5. Responsiveness

- ***RQ.1.5** Can energy consumption be lower when the system response time is short, allowing the task to be completed more quickly?*

Systems are always expected to have short time responses. An application is considered unresponsive in case of taking more than 200 ms to respond to a user event [112]. Responsiveness can be affected by network requests, database accesses, file I/O, and image processing that can become a bottleneck in software system performance [113,114]. Therefore, connectivity and I/O operations can have a dramatic impact on PHR tasks and, indirectly, affect on its responsiveness. From the results of the experiments, the more responsiveness, the more power consumption. These findings confirmed previous studies on the trade-off between energy efficiency and responsiveness [115]. That was the case of the processor ($r_{s}=0.268,\;p=0.013$) and the power supply ($r_{s}=0.246,\;p=0.023$).

NoMoreClipBoard spent high amounts of power in the processor and power supply when performing task 11, check medications. This PHR presents all the information at once in the profile, receiving the highest usability score in this principle of design. In this sense, the sooner the medical data is available, the better responsiveness. However, requesting all the data in a short period of time can lead to a high peak of power consumption. On the other hand, if the PHR may have to be idle to receive the data from the server, the total amount of power spent during the task can be also remarkable. As an example, in PatientsLikeMe, when performing task 4 check profile, there is a wall of news when accessing to the profile. In this sense, the responsiveness of PatientsLikeMe is lower since the wall is loaded as is navigated. Notice that the responsiveness and the peak power consumption were lower in PatientsLikeMe as compared with the rest of the sample elements. Thus, there should be a trade-off when implementing this principle between peak power (NoMoreClipBoard) and total power (PatientsLikeMe) consumption required to perform a task.

## 4. Conclusions and Future Work

This study investigated the relationship between energy consumption of the main PC components and usability assesments of GUIs, particularly in web-based PHRs. A set of 20 typical tasks have been conducted to evaluate the usability according to 14 heuristics and the energy consumption measured with the EET equipment. Results showed that there is a weak relationship between usability and energy efficiency, with significant correlation of some usability principles with PC components consumption. Consistency, task migratability, observability, recoverability, and responsiveness were the usability principles that had the strongest correlation with energy consumption. In some cases these correlations were positive in others negative.

This study might have several limitations: (i) The focus on web-based PHRs only instead of different range of software applications. However, a specific type of applications was chosen with the aim to setup the typical tasks to be followed for the usability and energy consumption evaluation. (ii) The energy consumption results are related to a PC with particular properties, replicating this study in another machine might have different results. It is worth noting that the same PC has been used in the evaluation of the five selected web-based PHRs, which showed the different level of energy consumption of each PHR. (iii) Using the Dix principles of design [44] instead of other heuristic to evaluate usability might have impacted the results. Nevertheless, these principles are very well known and applied in web development community. Since this work focused on web-based PHRs, these principles were adequate for evaluating usability. (iv) Only one auditor evaluated the usability of the tasks one by one, employing the Dix principles of design. According to Nielsen, when there is only one auditor the proportion of usability problems covered in the assessment is around 50%. This percentage presents a maximum when the number of auditors are between 4 and 5, which in that case is around 70% [116]. (v) HealthVault was closed in November 2019. This fact makes it difficult to repeat the experiment again with the same PHR. It is worth noting that not many PHRs were found on the Internet that met the inclusion and exclusion criteria. An interesting analysis of the reasons why the PHR was not successful would provide information on the main factors influencing the adoption of a PHR. In any case, the experiment was carried out before the above date. Therefore, the results relating to HealthVault contributed to the objective of the research, and were described in the paper.

As opposed to previous research [117], the correlations obtained between usability assessments of GUIs and power consumption in client applications led to conclude that the posed hypothesis in this study might be plausible. That is to say, improving usability of the web portals by considering some of the heuristics presented in the paper may enhance energy consumption as well. The significant correlations of this study allowed to accept or reject the hypothesis, answering more precisely the main research question (i.e., RQ.1). As for RQ.1.1, a higher energy demand was found when Consistency scores were higher. In that case, the hypothesis under study could not be confirmed. As for RQ.1.2, a low energy demand was found when the scores on Task migratability heuristics were high, thus confirming the hypothesis. As indicated in RQ.1.3, giving the feeling that the system was running smoothly generated a higher energy consumption, rejecting in this case the hypothesis of this work. It should be noted that the correlation calculated was close to being significant ($r_{s}=0.208,\;p=0.055$). As for RQ.1.4, energy consumption was lower when the perception of the Recoverability heuristic was high, thus confirming the hypothesis of the study. Finally, in the case of RQ.1.5, when the feeling of speed in receiving the answers given by the system was high, there was a higher energy consumption, thus discarding the hypothesis. At this end, a tradeoff between usability and energy expense must be considered. Some examples are exposed briefly below. Moreover, some design patterns concerning the findings in the paper are presented in Table 5.

- **Consistency.** The single sign-on mode was a promising solution to exit the PHRs and improve usability. On the other hand, logging out from several services at once may produce a peak of power consumption. This large amount of energy could be generated not only from the server side, but also in the client computer. Logout processes should be simplified to achieve energy efficiency.
- **Task migratability.** Autocomplete feature was a good example that produced energy efficiency together with good usability of the web portal. However, this functionality was not present in all the PHRs. In order to improve usability and power consumption, this functionality should be implemented whenever is possible. A particular procedure to perform good predictions should be developed. In addition, usability should be born in mind when showing the results.
- **Observability.** A good usability is related to the use of traditional widgets such as loading icons, progress bars and scrollbars. All of them are well-known graphic components that enhance usability. However, this is not at zero energy cost. These elements should be updated with the design of new visual interfaces for the same or greater functionality. Moreover, these new interfaces should be simpler to use and energy efficient.
- **Recoverability.** Keeping the writing in the forms, when moving to another page enhances usability. This feature avoids to type again the content when coming back to the form, and allows to finish the task quicker. The implementation of this functionality should focus on sustainability to keep the text in the forms with the less amount of energy possible.
- **Responsiveness.** There must be a good balance between simple and overloaded interfaces to deal with this principle properly. The simpler the interface, the faster the web page will load. However, more time or more display changes may be needed to accomplish a task. On the other hand, a complex interface may demand more energy while it is being processed.

More studies should be carried out to develop a common understanding on the topic of the paper. In this manuscript the evaluation of energy consumption and usability was focused on the client side. An approach was proposed to investigate how usability can impact on the energy consumption of a PC when running a client application. However, the server side also has a significant impact on the environment. Renewable energies may not be used commonly, or resources may not be well managed in data centres [118]. In some websites such as The Green Web Foundation [119] data on server energy practices are collected. This information could serve as a basis for studying the server side in future work.

New heuristics could be defined for a new framework specially designed for usability evaluations in the PHRs. In that case, the literature should be analysed in detail and a protocol for using and validating the heuristics should be proposed. Another future work could be the design of a software requirements catalogue based on the usability and energy consumption of the client applications. The contribution of a software requirements catalogue on both topics could help researchers and practitioners in developing energy-aware software [8]. One way of addressing these tasks could be to carry out two separate comprehensive studies of usability assessment of GUIs and energy spent on client applications. The results of both studies could be compared with the aim of detecting further correlations between usability evaluations and energy consumption in client applications. Another possibility for future work could be the development of a framework based on graphical components in the GUIs that improve usability. The impact of the aforementioned components on energy consumption could be analysed, providing an energy consumption score based on the components placed on the web portals. This framework would have a double objective. On the one hand, it would allow auditing the usability and energy consumption of the client applications. On the other hand, it would help the technicians in the development of the aforementioned tools.

## Figures and Tables

**Table 1:** Features of the selected PHRs.

PHR	Website	Target	Developer	Certications	Relevant features
HealthVault	healthvault.com	General users	Microsoft	Not found	(1) In different languages; (2) Closed on November 2019
HealthVet	myhealth.va.gov	USA veterans	VA dept.	Not found	More than 18 million pages of health information
PatientsLikeMe	patientslikeme.com	General users	Private company	Not found	(1) More than 2800 conditions tracked; (2) Social network oriented; (3) Daily mood scoring
NoMoreClipBoard	nomoreclipboard.com	General users	Private company	ONC-ACB	(1) Approved for both outpatient and inpatient use; (2) Overelaborate GUI; (3) Thematic icons to organize the information
Health Companion	healthcompanion.com	General users	Private company	ONC-HIT & GeoTrust	(1) Health information stored securely in accordance with US federal regulations; (2) Finance management; (3) Minimalist GUI

**Table 2:** Typical activities to be done in a PHR.

#	Task
Activity 01:	Sign up
Activity 02:	Login
Activity 03:	New profile
Activity 04:	Check profile
Activity 05:	Provide access to external users
Activity 06:	Complete family history
Activity 07:	New medication
Activity 08:	Include allergy
Activity 09:	Incorporate vaccine
Activity 10:	New disease
Activity 11:	Check medications
Activity 12:	Print report
Activity 13:	Check glucose evolution
Activity 14:	Information of conditions search
Activity 15:	Export health information
Activity 16:	Arrange appointments and enter medication reminders
Activity 17:	Contact/propose suggestions
Activity 18:	Read privacy policy
Activity 19:	Logout
Activity 20:	Password recovering

**Table 3:** Spearman’s correlations between power in watts and usability evaluations (Likert scale).

	Hard Disk		Graphics Card		Processor		Monitor		Power Supply	
	$r_{s}$	*p*	$r_{s}$	*p*	$r_{s}$	*p*	$r_{s}$	*p*	$r_{s}$	*p*
Predictability	−0.017	0.875	−0.191	0.077	0.057	0.600	−0.005	0.958	0.079	0.469
Synthesizability	−0.091	0.404	0.006	0.958	−0.056	0.606	0.079	0.469	−0.068	0.533
Familiarity	−0.038	0.728	0.015	0.888	0.025	0.820	−0.075	0.491	0.041	0.709
Generalizability	−0.018	0.866	−0.057	0.600	−0.112	0.306	0.054	0.621	−0.139	0.202
Consistency	0.114	0.298	0.147	0.178	0.238 *	0.027	0.246 *	0.023	0.254 *	0.019
Dialog initiative	0.043	0.696	0.058	0.598	0.075	0.491	0.119	0.276	0.093	0.393
Multi-threading	0.065	0.550	0.000	0.999	−0.033	0.766	0.124	0.254	−0.047	0.666
Task migratability	−0.024	0.829	−0.191	0.079	0.212 *	0.050	−0.024	0.827	0.237 *	0.028
Substitutivity	−0.012	0.911	−0.136	0.213	0.007	0.949	0.039	0.719	0.012	0.913
Customizability	0.026	0.811	−0.157	0.149	−0.066	0.543	−0.061	0.576	−0.070	0.525
Observability	−0.153	0.159	−0.151	0.166	0.151	0.164	0.208	0.055	0.127	0.242
Recoverability	−0.279 **	0.009	−0.224 *	0.038	−0.050	0.649	−0.179	0.099	−0.113	0.300
Responsiveness	0.026	0.814	0.156	0.151	0.268 *	0.013	0.038	0.727	0.246 *	0.023
Task conformance	−0.125	0.250	−0.112	0.304	0.076	0.488	−0.088	0.421	0.029	0.794
Global	−0.078	0.474	−0.133	0.222	0.107	0.326	0.056	0.610	0.091	0.404

* The correlation is significant at the 0.05 level (bilateral); ** The correlation is significant at the 0.01 level (bilateral).

**Table 4:** The Split-Half Reliability coefficients.

PHR	Coefficient
HealthVet	0.89
HealthVault	0.84
NoMoreClipBoard	0.93
PatientsLikeMe	0.85
Health Companion	0.82

**Table 5:** Design patterns.

Heuristic	Pattern	Task	PHR
Consistency	Single sign-on mode	Login	HealthVault
Task migratability	Autocomplete	Information of conditions search	PatientsLikeMe
Observability	Loading icons with linear behaviour	Provide access to external users	Health Companion
Recoverability	Keep the writing in the forms	Contact/propose suggestions	NoMoreClipBoard
Responsiveness	Well balanced GUI	Check profile	HealthVet

## Data Availability

Publicly available datasets were analyzed in this study. This data can be found here: https://umubox.um.es/index.php/s/DBPrwlgvfMOGTp4.

## Abbreviations

The following abbreviations are used in this manuscript:

AHIMA	American Health Information Management Association
EC	Exclusion Criterion
EET	Energy Efficient Tester
FEETINGS	Framework for Energy Efficiency Testing to Improve Environmental Goals of
	the Software
GUI	Graphical User Interface
HCI	Human-Computer Interaction
IC	Inclusion Criterion
IS	Information System
PC	Personal Computer
PHR	Personal Health Records
PRISMA	Preferred Reporting Items for Systematic reviews and Meta-Analysis
SEA	Software Energy Assessment
